# An Audit of Opportunistic Lung Cancer Screening in a Canadian Province

**DOI:** 10.1177/21501327211051484

**Published:** 2021-10-19

**Authors:** Victoria Linehan, Scott Harris, Rick Bhatia

**Affiliations:** 1 QEII Health Sciences Center, Halifax, NS, Canada; 2 Health Sciences Centre, St. John’s, NL, Canada

**Keywords:** lung cancer, screening program, incidental findings, screening population

## Abstract

**Objectives:**

Lung cancer is a leading cause of cancer-related death in Canada. Early detection can improve outcomes and despite recommendations from the Canadian Task Force on Preventive Health Care to screen patients who are 55 to 74 years old and have a 30+ pack-year history, formal screening programs are rare in Canada. Our goal was to determine if screening is being performed in a representative Canadian population, if recommendations are being followed, and how screening impacts lung cancer stage at diagnosis and prognosis.

**Methods:**

A retrospective chart review was performed to identify patients either screened for lung cancer or imaged due to lung cancer symptoms in Eastern Newfoundland between 2015 and 2018. Age, smoking history, screening modality, diagnosis, cancer stage, and mortality were recorded.

**Results:**

Under 6.0% of the eligible population were screened for lung cancer with only 28.13% meeting age and smoking criteria and being screened appropriately with low-dose CT. However, 70% of patients that had lung cancers found by screening met age and smoking screening criteria. While lung cancer detection rates were similar, screening detected cancer in patients at an earlier stage (50% Stage 1) compared to patients who were not screened (20% Stage 1). Patients who were screened had an improved prognosis.

**Conclusions:**

Physicians are opportunistically screening for lung cancer, but not consistently following screening guidelines. As screening is sensitive, leads to earlier stage diagnosis, and has a mortality benefit, implementation of an organized screening program could increase quality assurance and prevent many lung-cancer related deaths.

## Introduction

Lung cancer is the leading cause of cancer-related death in Canada.^1^ Approximately 85% of lung cancer cases can be attributed to a smoking history and heavier use associated with greater risk.^2^ Mortality is predicted by the stage of cancer at diagnosis and in Canada 49% of lung cancers are diagnosed at Stage 4,^3^ with earlier detection resulting in more favorable outcomes.^2,4-7^ Early detection of disease may be achieved via screening programs. In 2014, the Canadian Task Force on Preventive Health Care (CTFPHC) recommended screening using 3 annual LDCT scans in high-risk individuals—those aged 55 to 74 who are current smokers or quit less than 15 years ago with a 30+ pack-year history.^2^ These recommendations were based on the National Lung Screening Trial (NLST), one of the first to assess low-dose CT (LDCT) for lung cancer screening, which found a 20% mortality benefit over screening with radiography.^5^ More recently, the Pan-Canadian Early Detection of Lung Cancer (PanCan) study, demonstrated that screening with LDCT can identify more cancers at earlier stages.^6^

In Canada, provincial government organizations are responsible for establishing cancer screening programs to ensure that guidelines are being followed by physicians and that programs are cost-effective for our publicly funded healthcare system. Despite the promising research on the benefits of screening and the CTFPHC recommendations, only 1 province has a formal organized Canadian lung cancer screening program, which was recently implemented. In lieu of a formal program, it is not yet known whether physicians in the Canadian province of Newfoundland and Labrador are opportunistically screening patients outside of an organized program and how this would affect cancer detection in our population. A recent environmental scan by the Canadian Partnership Against Cancer reported that opportunistic screening is occurring in 6 provinces, but rates of screening have not yet been determined.^8^ It is likely that opportunistic screening rates are low since 79.4% of lung cancer cases in Nova Scotia were detected as a result of symptomatic presentations^9^ and 44% of incident lung cancer cases in Ontario presented to the ER within a week of their diagnosis.^10^ Limited screening would also be consistent with the fact that 69% of lung cancers being Stage 3 or 4 at diagnosis in Canada.^3^

Newfoundland and Labrador has an age standard incidence and mortality of lung cancer of 75.1 cases and 57.4 deaths per 100 000, respectively.^11^ This is comparable to the median of 72.1 cases and 53.9 deaths per 100 000, respectively, for Canadian provinces and territories.^11^ Barriers to healthcare in Newfoundland and Labrador are similar across Canada. There is limited access of health services to patients living rurally, however we have more patients in rural areas (34.3%) compared to Canada as a whole with 18.9% of patients living rurally.^12^ There is lack of family physicians to provide care in urban and rural areas that is similar between our province (12.5%) and Canada as a whole (18.9%).^13^ However, all provinces in Canada, including Newfoundland and Labrador, have organized colon, cervical, and breast cancer screening programs and have the capacity to develop an organized lung cancer screening program. Such a program could be cost-effective within the publicly-funded Canadian healthcare system and potentially cost-saving should noncurative cancer treatments be incorporated into predictive models.^4,14^

Therefore, our goal was to determine if opportunistic lung cancer screening is being performed in Newfoundland and Labrador, a Canadian province that is representative for lung cancer incidence and mortality, and if so, whether screening protocols are followed without an organized program to ensure quality assurance, and if there are measurable risks or benefits for screening in our patient population that could support implementation of organized screening programs in other Canadian provinces.

## Methods

We performed a retrospective review of all patients that underwent opportunistic lung cancer screening in the Eastern Health region of Newfoundland and Labrador (catchment area of around 300 000 patients) between January 2015 and December 2018. Our study population is 60% of the province’s population with a similar distribution of age and sex.^15^ Patients were identified using mPower Clinical Analytics (Nuance, Burlington, MA) to search the PACS database for imaging reports that contain key terms. To look for opportunistic screening studies, we used the terms “lung cancer,” “screening,” “family history,” “pack-years,” and “smoker/smoking” with filters for the study period, non-contrast chest CT, and chest radiograph (XR). To compare outcomes between patient who had lung cancer found by screening versus those who were not, we also identified symptomatic patients who were imaged to rule out lung cancer by using the search terms “lung cancer,” “malignancy,” “new cough,” “dyspnea,” hemoptysis,” and “weight loss” with the same filters as previous. Together, these searches returned 7648 unique reports for review. Each of these reports were read to determine if they met criteria for our study. We removed 1463 scans that were performed as a follow-up on previous imaging and 4382 reports that met exclusion criteria: previous lung or breast cancer, active malignancy or metastatic disease, vague or no indication provided, imaging for another disease process, and screening for other reasons. Finally, we separated studies into Screening and Diagnostic groups. The Screening group (534 reports in 506 patients) was defined as asymptomatic patients whose indications for imaging was either lung cancer screening, a family history of lung cancer, and/or smoking history. The Diagnostic group (1269 reports in 1266 patients used to identify 62 patients with lung cancer) were patients who were diagnosed with lung cancer not through screening but because they were evaluated for 1 of more of the symptoms of cough, dyspnea, chest pain, weight loss, and hemoptysis, or had a clinical suspicion of lung cancer.

For the Screening group, imaging reports were reviewed to determine age, sex, smoking history, and modality (XR, CT, LDCT, or ultra-low dose CT [ULDCT]) used to determine whether they met current lung cancer screening recommendations. We recorded results of screening and any additional imaging such as previous or repeat screening and follow-up imaging on relevant or incidental findings up until January 2021. We also checked prior records to ensure our patients in our Diagnostic group who had been diagnosed with cancer had not been previously screened. For the Diagnostic group, we recorded age, sex, indication for imaging, and smoking history as well as findings from initial and follow-up reports. In both groups, if the report indicated a suspicion for malignancy, electronic medical charts were reviewed for tissue diagnosis, stage, treatment intent, and mortality. These outcomes were used to compare the patients in the Screening and Diagnostic groups who had been diagnosed with lung cancer to determine whether screening for lung cancer improves stage at diagnosis and survival. Additionally, charts were reviewed for all Screening group patients to determine if any lung malignancy was missed by screening.

Statistical analyses were performed using linear regression for time effect plots, Fisher’s exact tests or χ^2^ for proportional data, Kolmogorov-Smirnov test for frequency distributions, unpaired *t*-tests for continuous data, and log-rank tests for survival data. Analyses were performed in GraphPad Prism 6 and R software. Data were expressed with standard error and a *P*-value of <.05 was considered significant.

Ethics approval was obtained from Memorial University of Newfoundland Health Ethics Research Board and permission was obtained from data custodians to review patient records.

## Results

**Figure 1.**
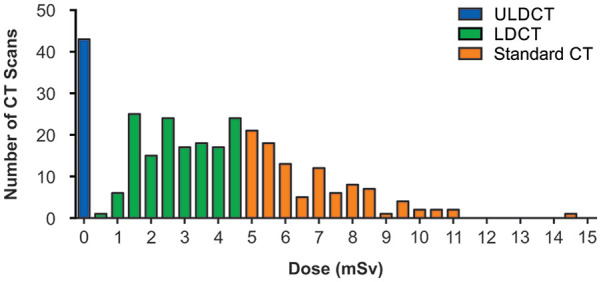
Doses of CT scans used for screening. LDCT, low-dose CT; ULDCT, ultra lose-dose CT.

**Figure 2.**
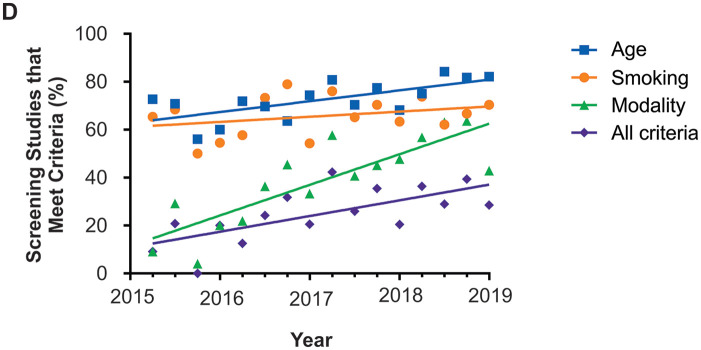
The number of studies meeting all modality and age criteria, as well as studies meeting all criteria increased over time.

We estimated that 8421 people would be eligible for lung cancer screening based on CTFPHC recommendations in our catchment area based on age demographics, smoking habit, and proportion of smokers meeting the criterion based on our data.^16,17^ As we only identified 506 screened patients, screening is likely occurring in our province on a limited basis, this number representing only 6.0% of the estimated eligible population being opportunistically screened. To determine appropriateness of screening, we next assessed whether these patients met CTFPHC recommendations: patients who were 55 to 74 years old, current or ex-smokers (quit <15 years ago) with 30+ pack-years, and screened using LDCT. There were 51 patients with no documented smoking history and were excluded from this analysis (Table 1). Only 28.13% of patients met all CTFPHC recommendations, while a minority (8.79%) met none (Table 1). Although two thirds of patient met age or smoking criteria individually (Table 1) only 50.66% of patients met both. Many low-risk patients were screened as 9.49% were <50 years old, 3.95% were non-smokers, and 23.5% of smokers had <30 pack-years. Only 40.12% of patients were screened with ULDCT or LDCT, while the remainder were screened with standard-dose CT or XR (Table 1). There was great variability in doses used for screening CT ranging from 0.14 to 14.68 mSv (Figure 1). Family history was also provided on requisitions as an additional risk factor for 15.22% of patients and they were screened at younger ages (59.51 ± 0.62 vs 62.39 ± 0.46, *P* = .0002). There was a significant increase in the number of studies meeting all criteria over time (*P* = .0033; Figure 2), as well as studies using the appropriate modality (*P* < .0001) and age group (*P* = .0045) but there was no change in the number of studies that had appropriate smoking history over time (*P* = .2479; Figure 2).

**Table 1.** Appropriateness of Lung Cancer Screening.

Individual CTFPHC criteria for each patient^a^	Factors for lung cancer screening				Patients meeting each criterion (55-74 years old)	
	Age	60.81 ± 9.08 years old			364	71.94%
	Smoking history*	Smoking history*			Patients meeting criterion (30+ pack-years)	
	Modality	36.05 ± 21.11 pack-years			298	65.49%
		Modality			Patients meeting criterion (LDCT/ULDCT)	
		XR	113	22.33%	203	40.12%
		CT	190	37.55%		
		LDCT	160	31.62%		
		ULDCT	43	8.50%		
Total number* of CTFPHC criteria met for each patient^b^	None				40	8.79%
	One Criterion				126	27.69%
	Two Criteria				161	35.38%
	Three Criteria				128	28.13%

Abbreviations: LDCT, low-dose CT; ULDCT, ultra lose-dose CT.

a The number of patients who were screened for lung cancer and met the Canadian Task Force on Preventive Health Care (CTFPHC, 2016) individual criteria for age (55-74), smoking history (at least 30 pack-years; current smoker or quit within the past 15 years), and modality (LDCT or ULDCT).

b Total number of CTFPHC criteria that were met by each patient during the study period.

* Fifty-one patients did not have documented smoking history and so percentages were calculated from total patient population of 455.

**Figure 3.**
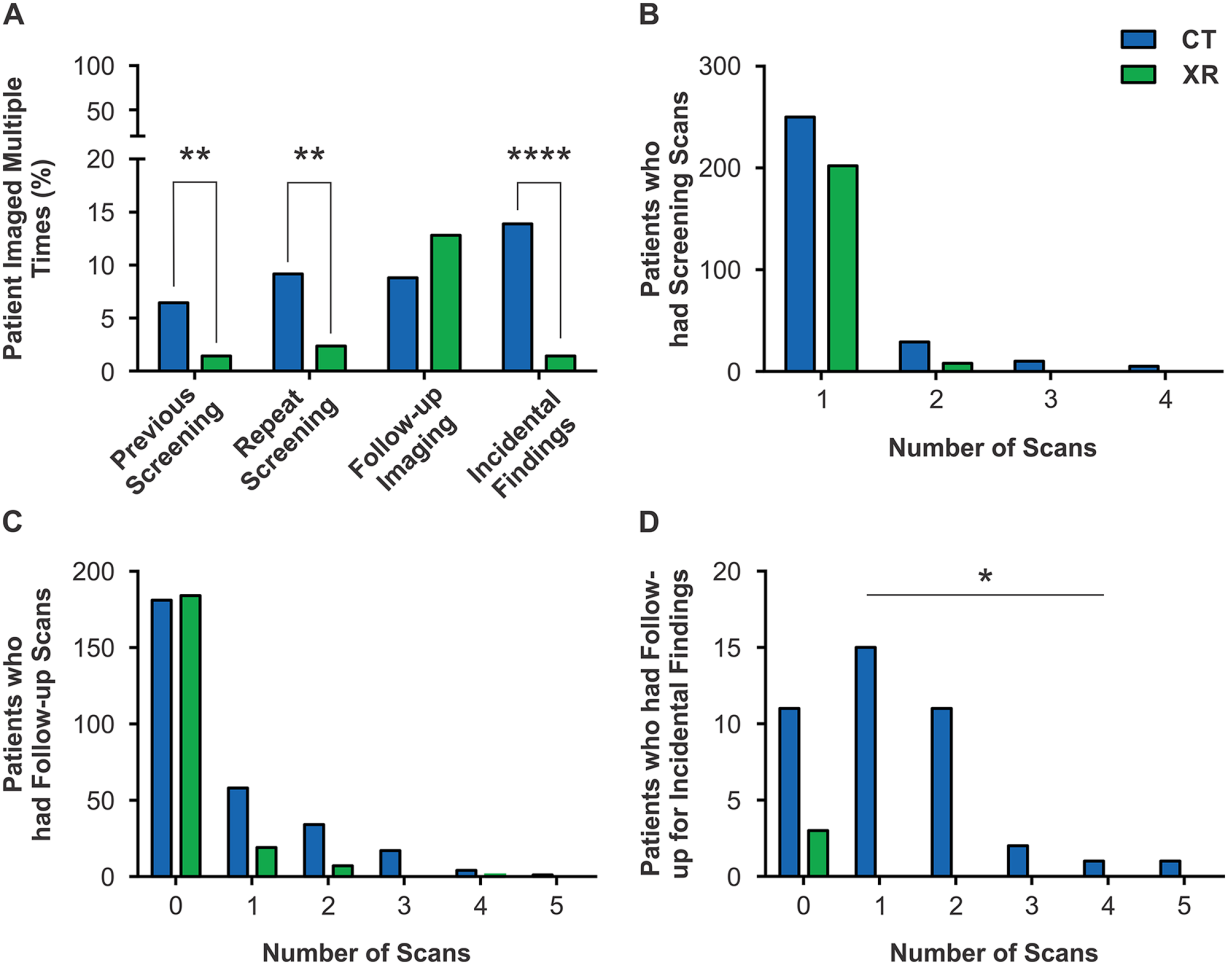
Additional imaging on patients screened for lung cancer: (A) the proportion of patients that underwent repeat screening, required follow-up imaging for an abnormality on their screening study, and had incidental findings on their screening study. (B) The total number of screening studies per patient. (C) The number of follow-up studies required for findings worrisome for malignancy per patient. (D) The number of scans required for incidental findings. *P < .05. **P < .01. ****P < .0001.

The CTFPHC recommends annual screening for 3 consecutive years; however, only 10.67% of patients were screened multiple times with 45.16% meeting age, smoking, and LDCT screening criteria (Figure 3A) and 1.98% were screened 3 times with only 33.33% meeting criteria (Figure 3B). Repeat screening was more likely if patients were imaged by CT at any dose (previous screening: *P* = .0068, repeat screening: *P* = .0015; Figure 3A). 10.47% of patients required follow-up imaging for pulmonary abnormalities found during screening, and neither the number of patients (*P* = .1848) nor the number of follow-up studies required (*P* = .9307) differed between any dose CT and XR (Figure 3A and C). However, any dose CT screening detected significantly more nodules in (37.97%) than XR (1.69%; *P* < .0001). Similarly, any dose CT screening revealed significantly more incidental findings (*P* < .0001; Figure 3A) and required more follow-up scans (*P* = .0278; Figure 3D) than XR. In the 33 patients that had incidental findings, 3 patients had significant findings of a hepatocellular carcinoma, thyroid nodule requiring hemithyroidectomy, and thymoma.

Patients were followed for 2 to 5 years after screening and no patients screened negative with any dose CT developed lung cancer while 3 patients screened negative with XR were diagnosed with lung cancer between 3.52 and 4.31 years after screening. There were 2 false positives—1 screened by LDCT that had a negative lavage and 1 screened by XR that had a negative tissue diagnosis following a lobectomy. There were also 2 cases of lymphoma diagnosed because of screening.

**Figure 4.**
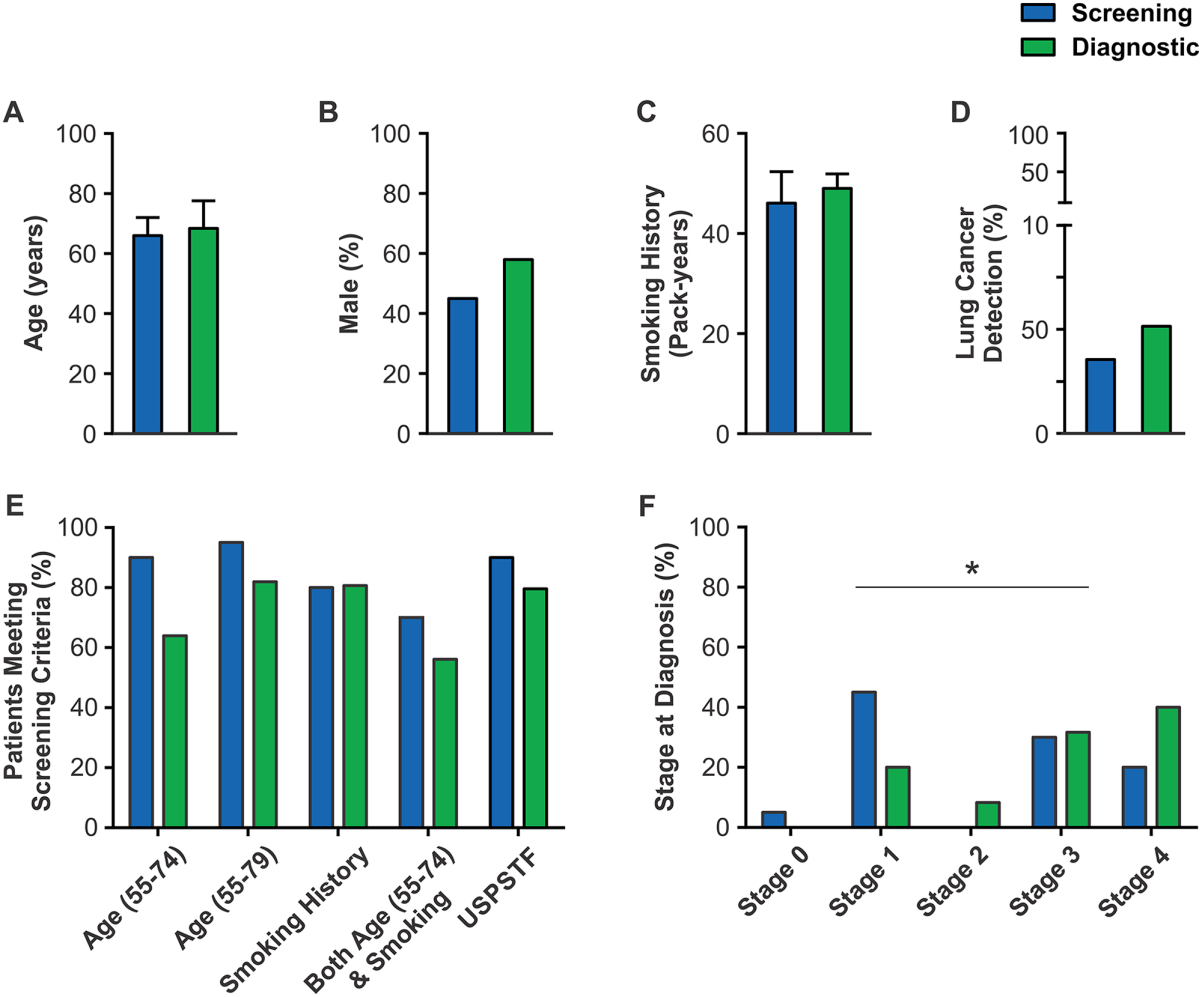
Comparison of patients diagnosed with lung cancer through screening (Screening group) or compared to those who were not screened (Diagnostic group): (A-C) There is no difference in age (A), sex (B), or smoking history (C) in lung cancer patients from the Screening or Diagnostic groups. (D) There was no significant difference in the detection rate of lung cancer. (E) The percentage of patients who met screening criteria in the Screening group, and those who would have met criteria if they had been screened in the Diagnostic group. (F) Patients screened for lung cancer were diagnosed at an earlier stage. *P < .05.

Finally, we compared asymptomatic patients who were diagnosed with lung cancer due to screening (Screening group; 20 patients) and symptomatic patients who were imaged to rule out malignancy (Diagnostic group; 62 patients). There were no differences in age, sex, or smoking history between groups (Figure 4A-C). Of these patients, 70.00% of the Screening group and 56.14% of the Diagnostic group met CTFPHC screening recommendations (Figure 4E). Therefore, lung cancer would have been missed in 30.00% of the patients in the Screening group if the CTFPHC recommendations were followed. Recently, the US Preventive Services Task Force (USPSTF) expanded lung cancer screening criteria to include patients who are 50 to 80 years old and currently smoke or quit <15 years ago with a 20+ pack-year history.^18^ Applying these criteria to our lung cancer patients, 90.00% and 79.66% of the Screening and Diagnostic groups, respectively, met USPSTF guidelines, meaning that only 10% to 20% of lung cancers would have been missed using their screening guidelines in our population (Figure 4E).

**Figure 5.**
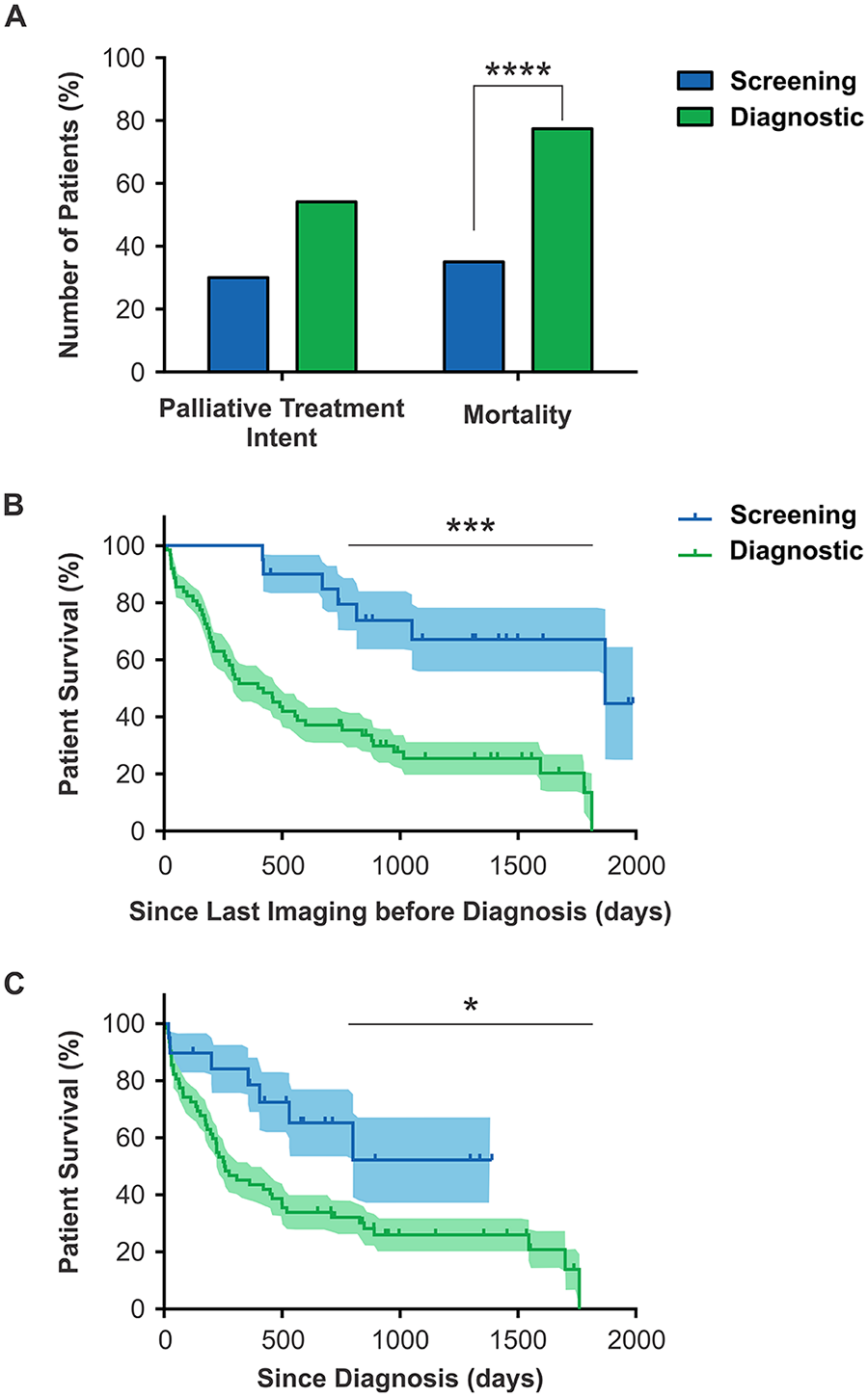
Survival rates in patients with lung cancer were significantly lower in those who were not screened: (A) There was no difference in the proportion of patients treated with palliative intent (left) but a significantly higher proportion of patients died (right) who were not screened. (B) A survival curve for lung cancer patients measured from the day of the last imaging that indicated suspected malignancy. (C) A survival curve for lung cancer patients measured from the day of their diagnosis. *P < .05. ***P < .001. ****P < .0001.

There was no significant difference in the lung cancer detection rate (Screening: 3.46% vs Diagnostic: 5.15%; *P* = .2085, Figure 4D) nor the distribution of tissue diagnoses between groups. However, there was a difference in the stage of lung cancer at diagnosis—patients screened for lung cancer were diagnosed at earlier stages with 50.00% at Stage 1 (including 1 patient diagnosed at Stage 0), while 80.00% in the Diagnostic group were at Stage 2 to 4 (*P* = .0185; Figure 4F). Despite the difference in stage, there was no difference in the proportion of patients initially treated with palliative versus radical intent between the groups (*P* = .0748) but there was significantly higher mortality in the Diagnostic group (*P* < .0001, Figure 5A). Accordingly, there was significantly reduced survival from the last imaging that raised suspicion for lung cancer (*P* = .0001; Figure 5B) and the interval from diagnosis (*P* = .0227; Figure 5C).

## Discussion

Despite the lack of an organized program by a provincial body to ensure screening uptake and quality assurance, lung cancer screening is being performed in the Canadian province of Newfoundland and Labrador on a limited basis as we estimated less than 6% of the eligible population is being screened. Moreover, half of patients being screened do not meet CTFPHC recommendations. Nevertheless, patients who were screened were diagnosed at earlier stages and had a survival benefit over those who were not. This study supports lung cancer screening programs.

Our first aim was to determine whether lung cancer screening was following recommendations by the CTFPHC.^2^ Only half of patients that were being screened had the appropriate age and smoking history. The CTFPHC recommendations, like those from American medical societies,^19-22^ are based on the NLST.^5^ Most recommend that patients have a 30+ pack-year history and be a current smoker or ex-smoker of at least 15 years, except for the new USPSTF guidelines which have reduced this to 20+ pack-years.^18^ While all recommend a start time of 55 years old, the age to stop screening is 74 for the CTFPHC but can vary up to 80 years old.^18,22^ This expanded age range would only include an additional 2.77% of our screening patients. On the other hand, several societies recommend screening younger patients if they have another risk factor such as family history, chronic lung disease, or exposure to carcinogens.^19,22^ In our study, some screening requisitions included family history and these patients were indeed screened at a younger age, but their average age was still within the recommended range for screening. Therefore, differences between lung cancer screening recommendations that were available during our study period cannot explain why half of the patients in our Screening group were not at high-risk based on age and smoking history. There may have been increasing awareness of screening guidelines over time since the proportion of patients screened in the appropriate age range significantly increased throughout our study period.

Beyond patient demographics, LDCT is the standard recommendation for lung cancer screening.^19-22^ In our study, LDCT was used in only 40.12% of screening studies. Without a formal screening program, protocoling by radiology staff and residents would be inconsistent. However, we did see a shift in screening modality over the study period, which perhaps was due to increasing awareness of lung cancer screening recommendations. This could be further improved with the implementation of a formal screening program.

Since the NLST screened patients for 3 consecutive years,^5^ the CTFPHC recommends only 3 annual scans for lung cancer screening.^2^ Other guidelines state that annual screening should be performed throughout the high-risk age range or until patients have been smoke-free for 15 years^19-22^; however, evidence is lacking to compare between these recommendations. Regardless, most patients in our study received only 1 screening scan, meeting neither recommendation.

Taken together, evidence-based guidelines are not being followed to identify high-risk patients, appropriately screen with LDCT, and then request follow-up screening. While there was significant improvement in adherence to recommendations over the study period, this has been a steady change rather than a sudden increase in response to publication of a new guideline.

While our study is limited by its retrospective design, smaller sample size, and lead time bias, we show significant benefits of lung cancer screening in our population, despite the lack of adherence to screening recommendations. The stage at diagnosis for our patients who were not screened is similar to the Canadian average,^3^ while our screening group was diagnosed at significantly earlier stages. In our study, 50% of lung cancers were identified by screening at Stage 1, which is comparable to the LDCT group (53%) in the NLST.^5^ A more recent trial for lung cancer screening, the PanCan study,^6^ had a higher proportion of early-stage cancers at diagnosis (77%), which may be due to their selection of higher-risk participants based on various predictor variables. As a single-arm study, PanCan could not compare mortality rates^6^ but the NLST showed a mortality benefit by screening with LDCT compared to XR.^5^ Retrospectively, we show that patients who were screened had a survival benefit over those who were not. While this could be due to a lead-time bias, lung cancers diagnosed at an earlier stage are more treatable and have a better prognosis.^23^ This data suggests a potential benefit for lung cancer screening in our province.

There are several potential harms of lung cancer screening. Screening with any dose CT has a high nodule detection rate, which often requires follow-up imaging.^6,24^ Nodule detection in our study was 23%, comparable to the NLST at 27%,^5^ while the PanCan detection rate was higher at 76%.^6^ Lung cancer detection rate was similarly higher in the PanCan study at 6.5%,^6^ followed by the NLST at 4.5%,^5^ and finally our study at 3.6%. These numbers appear to reflect the level of risk for each population studied, with PanCan selecting the highest risk patients,^6^ NLST selecting high-risk patients,^5^ and our population loosely following the NLST-based criteria. However, without trials to compare screening criteria, it is unknown if lung cancers are missed in lower-risk patients who fall outside selection criteria. In our study, 30% of patients who were diagnosed with lung cancer through screening did not meet CTFPHC/NLST criteria. With the PanCan inclusion criteria being stricter, our data suggest that adoption of either selection process may miss a significant number of lung cancer diagnoses. In contrast, applying the recent lung cancer screening recommendations from the USPSTF (2021) that included an expanded age range and a lower threshold for smoking history^18^ would have detected 90% of our screened patients who were diagnosed with lung cancer. Therefore, our findings support the use of the new USPSTF recommendations as a more sensitive screening program for lung cancer. Trials comparing PanCan, CTFPHC, and USPSTF guidelines on the outcomes of lung cancer detection, mortality, unnecessary interventions from incidental findings, and healthcare costs are required to implement the optimal population-based strategy to screen for lung cancer in Canada.

Other considerations of screening include incidental findings that may cause anxiety and lead to follow-up imaging for benign findings. While some recommend against looking for incidental findings on screening scans since less than 1% are significant,^25^ others report that 20% of screening scans will detect an incidental finding and about half of those will need follow-up.^24^ In our study, 41 patients (14%) who were screened by any dose CT had incidental findings and 30 patients had follow-up imaging with only 3 having medically actionable findings. This follow-up imaging adds additional costs to screening programs and has been previously factored into cost-effectiveness analyses that support lung cancer screening.^26^ While radiation exposure is also a potential harm of screening, evidence suggests that it is acceptable as the risk of lung cancer is 3 to 4 times greater than the risk of developing a cancer from radiation exposure.^27^ To further reduce this risk, ULDCT has also been shown to effectively detect nodules and cancers as an alternative to LDCT.^28^ There were no missed cancers in our CT screening group and 2 diagnosed patients were screened using ULDCT. It was noted however, in our population, CT dose was not consistent with some studies using either ULDCT, LDCT, or standard dose CT.

## Conclusion

In conclusion, our retrospective study is the first of this kind to report lung cancer screening practices in Newfoundland and Labrador and, despite its limitations, suggests a potential benefit of screening to diagnose lung cancer at earlier stages and improve survival. An organized provincial program could improve the quality assurance of screening, allowing for appropriate patient selection to increase cost-effectiveness, and ensure all patients at risk are being screened. Interestingly, the age and smoking history between our screening and diagnostic groups did not differ, and over half of patients that were diagnosed with lung cancer met screening guidelines and may have been diagnosed at an earlier stage with better survival outcomes.

The PanCan study has showed the effectiveness of screening a high-risk population for lung cancer,^6^ with favourable cost-effectiveness in our single-payer system.^4^ Our study supports the need for a provincial screening program and lessons learned that can be applied to other provinces. Namely, (1) opportunistic screening is occurring, but patients are not being selected according to guidelines and CT doses used for screening are variable, (2) 70% of lung cancers are detected if following CTFPHC guidelines which is improved to 90% when following the 2021 USPSTF guidelines, and (3) screening is detecting lung cancer at earlier stages with a mortality benefit. Therefore, implementation of a screening program with carefully evaluated screening criteria to balance lung cancer detection and costs could both increase appropriateness of screening and prevent lung-cancer related deaths.

## References

[1] Brenner DR Weir HK Demers AA , et al. Projected estimates of cancer in Canada in 2020. Can Med Assoc J. 2020;192(9):E199-E205. doi:10.1503/cmaj.191292 PMC7055947 32122974

[2] Canadian Task Force on Preventive Health Care. Recommendations on screening for lung cancer. CMAJ. 2016;188(6):425-432. doi:10.1503/cmaj.151421 26952527 PMC4818132

[3] Statistic Canada. Cancer in Canada: stage at diagnosis. Health reports. 2018. Accessed January 31, 2021. https://www150.statcan.gc.ca/n1/pub/82-003-x/2018012/article/00003-eng.htm

[4] Goffin JR Flanagan WM Miller AB , et al. Cost-effectiveness of lung cancer screening in Canada. JAMA Oncol. 2015;1(6):807-813. doi:10.1001/jamaoncol.2015.2472 26226181

[5] Aberle DR Adams AM Berg CD , et al. Reduced lung-cancer mortality with low-dose computed tomographic screening. New Engl J Med. 2011;365(5):395-409. doi:10.1056/NEJMoa1102873 21714641 PMC4356534

[6] Tammemagi MC Schmidt H Martel S , et al. Participant selection for lung cancer screening by risk modelling (the Pan-Canadian Early Detection of Lung Cancer [PanCan] study): a single-arm, prospective study. Lancet Oncol. 2017;18(11):1523-1531. doi:10.1016/S1470-2045(17)30597-1 29055736

[7] Byrne SC Barrett B Bhatia R. The impact of diagnostic imaging wait times on the prognosis of lung cancer. Can Assoc Radiol J. 2015;66(1):53-57. doi:10.1016/j.carj.2014.01.003 24931045

[8] Canadian Partnership against Cancer. Lung cancer screening in Canada: environmental scan; 2019. Canadian Partnership against Cancer. https://www.partnershipagainstcancer.ca/topics/lung-cancer-screening-environmental-scan-2018/

[9] Suhail A Crocker CE Das B Payne JI Manos D. Initial presentation of lung cancer in the emergency department: a descriptive analysis. CMAJ Open. 2019;7(1):E117-E123. doi:10.9778/cmajo.20180061 PMC6404960 30808631

[10] Habbous S Khan Y Langer DL , et al. The effect of diagnostic assessment programs on the diagnosis and treatment of patients with lung cancer in Ontario, Canada. Ann Thorac Med. 2021;16(1):81-101. doi:10.4103/atm.ATM_283_20 33680129 PMC7908893

[11] Canadian Cancer Society, Statistics Canada, Public Health Agency of Canada. Canadian cancer statistics: a 2020 special report on lung cancer. Heal Promot Chronic Dis Prev Canada. 2020;40(10):325-325. doi:10.24095/hpcdp.40.10.05 PMC7608932 33064075

[12] Statistics Canada. Canada goes urban. 2015. Accessed August 23, 2021. https://www150.statcan.gc.ca/n1/pub/11-630-x/11-630-x2015004-eng.htm

[13] Statistics Canada. Primary health care providers, 2019. 2020. Accessed August 23, 2020. https://www150.statcan.gc.ca/n1/pub/82-625-x/2020001/article/00004-eng.htm

[14] Cressman S Peacock SJ Tammemägi MC , et al. The cost-effectiveness of high-risk lung cancer screening and drivers of program efficiency. J Thorac Oncol. 2017;12(8):1210-1222. doi:10.1016/j.jtho.2017.04.021 28499861

[15] Statistics Canada. Census profile, 2016 census. 2016. Accessed August 23, 2021. https://www12.statcan.gc.ca/census-recensement/2016/dp-pd/prof/details/page.cfm?Lang=E&Geo1=HR&Code1=1011&Geo2=PR&Code2=10&SearchText=EasternRegionalHealthAuthority&SearchType=Begins&SearchPR=01&B1=All&GeoLevel=PR&GeoCode=1011&TABID=1&type=0

[16] Statistics Canada. Smokers, by age group. 2019. Accessed January 31, 2021. https://www150.statcan.gc.ca/t1/tbl1/en/tv.action?pid=1310009610

[17] Government of Newfoundland and Labrador. Population and demographics. 2020. Accessed January 31, 2021. https://stats.gov.nl.ca/Statistics/Statistics.aspx?Topic=population.

[18] Krist AH Davidson KW Mangione CM , et al. Screening for lung cancer. JAMA. 2021;325(10):962. doi:10.1001/jama.2021.1117 33687470

[19] McKee BJ Regis S Borondy-Kitts AK , et al. NCCN guidelines as a model of extended criteria for lung cancer screening. J Natl Compr Cancer Netw. 2018;16(4):444-449. doi:10.6004/jnccn.2018.7021 29632062

[20] Mazzone PJ Silvestri GA Patel S , et al. Screening for lung cancer. Chest. 2018;153(4):954-985. doi:10.1016/j.chest.2018.01.016 29374513

[21] Wender R Fontham ET Barrera E Jr , et al. American Cancer Society lung cancer screening guidelines. CA Cancer J Clin. 2013;63(2):107-117. doi:10.3322/caac.21172 23315954 PMC3632634

[22] Jaklitsch MT Jacobson FL Austin JHM , et al. The American Association for Thoracic Surgery guidelines for lung cancer screening using low-dose computed tomography scans for lung cancer survivors and other high-risk groups. J Thorac Cardiovasc Surg. 2012;144(1):33-38. doi:10.1016/j.jtcvs.2012.05.060 22710039

[23] Goldstraw P Crowley J Chansky K , et al. The IASLC lung cancer staging project: proposals for the revision of the TNM stage groupings in the forthcoming (seventh) edition of the TNM classification of malignant tumours. J Thorac Oncol. 2007;2(8):706-714. doi:10.1097/JTO.0b013e31812f3c1a 17762336

[24] Kucharczyk MJ Menezes RJ McGregor A Paul NS Roberts HC. Assessing the impact of incidental findings in a lung cancer screening study by using low-dose computed tomography. Can Assoc Radiol J. 2011;62(2):141-145. doi:10.1016/j.carj.2010.02.008 20382501

[25] van de Wiel JC Wang Y Xu DM , et al. Neglectable benefit of searching for incidental findings in the Dutch-Belgian lung cancer screening trial (NELSON) using low-dose multidetector CT. Eur Radiol. 2007;17(6):1474-1482. doi:10.1007/s00330-006-0532-7 17206426

[26] Black WC Gareen IF Soneji SS , et al. Cost-Effectiveness of CT screening in the National Lung Screening Trial. New Engl J Med. 2014;371(19):1793-1802. doi:10.1056/NEJMoa1312547 25372087 PMC4335305

[27] Rampinelli C De Marco P Origgi D , et al. Exposure to low dose computed tomography for lung cancer screening and risk of cancer: secondary analysis of trial data and risk-benefit analysis. BMJ. 2017;356:j347. doi:10.1136/bmj.j347 PMC5421449 28179230

[28] Huber A Landau J Ebner L , et al. Erratum to: performance of ultralow-dose CT with iterative reconstruction in lung cancer screening: limiting radiation exposure to the equivalent of conventional chest X-ray imaging. Eur Radiol. 2016;26(10):3653-3652. doi:10.1007/s00330-015-4192-3 27048537

